# Lipid-to-neutrophil ratios in predicting in-hospital outcomes in pulmonary thromboembolism

**DOI:** 10.34172/jcvtr.33254

**Published:** 2024-12-23

**Authors:** Neda Roshanravan, Erfan Banisefid, Samad Ghaffari, Sami Rassouli, Amirreza Naseri, Tohid Yahyapoor, Elnaz Javanshir, Sina Hamzezadeh

**Affiliations:** ^1^Cardiovascular Research Center, Tabriz University of Medical Sciences, Tabriz, Iran; ^2^Student Research Committee, Tabriz University of Medical Sciences, Tabriz, Iran; ^3^Research Center for Evidence Based-Medicine, Iranian EBM Center: A Joanna Briggs Institute Center of Excellence, Tabriz University of Medical Sciences, Tabriz, Iran; ^4^Tabriz USERN Office, Universal Scientific Education and Research Network (USERN), Tabriz, Iran

**Keywords:** Mortality, Neutrophil to high-density lipoprotein, Pulmonary thromboembolism, Prognosis

## Abstract

**Introduction:**

Acute pulmonary thromboembolism (PTE) is one of the leading causes of death and severe disability. Considering the impact of inflammation and lipid profile on prevalence and prognosis of deep vein thrombosis and PTE, this study was conducted to assess the predictive value of lipid-to-neutrophil count ratios for the short-term survival of PTE patients.

**Methods:**

This study is an analytical cross-sectional study. Data regarding the demographics, past medical history, vital signs, laboratory variables, and the outcomes of hospitalization were gathered from the Tabriz PTE registry. The receiver operating characteristics (ROC) curve and area under curve (AUC) were utilized for assessing the prognostic values. SPSS 26 was used for all of the statistical analysis.

**Results:**

The population of this analytical cross-sectional study consists of 547 PTE patients of which 41 patients (7.5%) died during hospitalization. There was a significant difference between death and survived groups regarding cholesterol (146.00[60.50] vs. 165.50[59.75]; p-value<0.01), LDL (80.00[48.00] vs. 102.00[52.00]; p-value<0.01), HDL (31.00[19.00] vs. 35.00[14.00]; p-value=0.04). Cholesterol/neutrophil*1000 with a cut-off value of 22.014 (sensitivity: 56.7%; specificity: 61.3%), LDL/neutrophil*1000 with a cut-off value of 10.909 (sensitivity: 69.3%; specificity: 51.9%) and HDL/neutrophile *1000 with a cut-off value of 4.150 (sensitivity: 61.9%; specificity: 58.1%) can predict short-term survival in patients with acute PTE.

**Conclusion:**

Based on our findings, patients with higher cholesterol/neutrophil, LDL/neutrophil, and HDL/neutrophil ratios have a better in-hospital prognosis and measurement of lipid-to-neutrophil ratio in the first 24 hours of hospitalization may be a valuable marker for determining the early prognosis of PTE. However, additional clinical studies are suggested for a more definitive conclusion.

## Introduction

 Pulmonary thromboembolism (PTE) is the third most common cause of cardiovascular mortality worldwide after stroke and myocardial infarction (MI).^1^ The annual incidence of PTE is approximately 300 000 to 600 000 cases in the United States (US) and Europe^2^ and it is increasing over the past 20 years.^3^ PTE imposes a significant economic burden on the healthcare system.^4^

 Risk stratification plays an important role in patients with acute PTE. Thrombolytic therapy or surgical embolectomy should be considered in patients with high-risk PTE.^5^ There are many clinical risk scores for evaluating PTE prognosis. The most common one is the Pulmonary Embolism Severity Index (PESI)^6^; beyond that, the availability of some blood parameters can also help us determine the PTE prognosis.^7,8^

 Neutrophils are one of the major components of leukocytes in the peripheral blood and play a significant role in thrombosis. It can be determined in a cheap and easily available way. Recent studies showed an increase in the blood level of neutrophils in patients with PTE.^9,10^ Also, total cholesterol (TC), high-density lipoprotein (HDL), low-density lipoprotein (LDL), and triglyceride (TG) are found to be associated with the incidence and prognosis of venous thromboembolism (VTE) and PTE.^11,12^ Based on pioneering investigations high serum HDL and TC levels are could be associated with a decreased risk of deep vein thrombosis (DVT). Studies also claim that high TG levels are associated with increased risk of DVT.^13^ HDL is also shown to have a negatively correlation with the onset of PTE.^14^ In addition, dyslipidemia is suggested to be correlated with PTE mortality. Higher initial serum HDL, LDL, and TC levels were reported to be associated with lower mortality rates in PTE patients.^15^

 Best of our knowledge, there is no study on the association between lipid-to-neutrophil ratios and the mortality of acute PTE. With these considerations, our study was conducted to assess the predictive value of lipid-to-neutrophil ratios for in-hospital mortality (IHM) in patients with acute PTE.

## Materials and Methods

 This study is an analytical cross-sectional study that was conducted between April 2019 and September 2022 at Shahid Madani Medical and Training Heart Hospital, affiliated with Tabriz University of Medical Sciences (TUOMS). The study process was reviewed and approved by the ethics committee of TUOMS, according to the declaration of Helsinki.

 The data for this research was retrieved from the Tabriz PTE registry. Informed consent was obtained from all subjects and/ or their legal guardian(s) who have participated in our study. Inclusion criteria were hospitalization with confirmed acute PTE diagnosis based on computed tomography (CT) pulmonary angiography by two expert radiologists, age ≥ 18, and availability of complete blood count data (CBC with *differential *measures of the number of each type of white blood cells). The exclusion criteria were chronic PTE. IHM was defined as any mortality during hospitalization due to PTE. Therefore, patients with mortality due to other causes were excluded from our sample. Information regarding the demographic characteristics, past medical history, vital signs (taken in the emergency unit), laboratory variables based on first fasting results after administration, and outcomes of hospitalization were collected and investigated ratios were calculated by dividing the lipid levels by the neutrophil counts. For example, cholesterol/neutrophil*1000 was calculated by dividing the serum TC levels (in mg/dL) by serum neutrophil counts per microliter multiplied in 1000.

###  Statistical analysis 

 The twenty-sixth version of SPSS Statistics was utilized for statistical analysis. The normality of the destitution of the numeric variables was assessed using the Kolmogorov–Smirnov test. Categorical variables are presented in number and percent. The Chi-square test was utilized for comparing these variables. Numerical parameters are reported in mean ± standard deviation (SD) or median and interquartile range (IQR), based on normality. The comparison between these variables was conducted using the independent sample t-test or Mann-Whitney U test. In addition, multivariate logistic regression analysis was conducted. Finally, the receiver operating characteristics (ROC) curve and area under curve (AUC) were utilized for the prediction of the IHM. In all applied statistics, 95% confidence intervals and a 0.05 level of significance for p-value were observed.

## Results

 Totally, 547 patients, including 253 males (46.2%) and 294 females (53.8%) included in this study. In Table 1 demographic characteristics and laboratory findings of patients who died due to PTE are compared to patients who survived it. There was a significant difference between death and survived groups regarding blood urea nitrogen (BUN) (*P *value < 0.01), creatinine (*P* value < 0.01), red cell distribution width (RDW) (*P* value < 0.01), lymphocyte (*P *value < 0.01), platelet (PLT) (*P *value = 0.04), systolic blood pressure (SBP) (*P *value < 0.01), cholesterol (*P *value < 0.01), LDL (*P *value < 0.01), HDL (p-value = 0.04), and HTN (*P *value < 0.01). In Table 2 results of logistic regression analysis for factors associated with PTE and IHM are shown. This analysis showed that none of these factors independently play a role in PTE and IHM association.

**Table 1:** The comparison of characteristics of the patients

**Characteristics **	**Death (n=41)**	**Alive (n=506)**	***P *** **value**
Age	77.00 [20.00]	71.00 [28.00]	0.07
Sex (male: female)	20 (48.8%): 21 (51.2%)	233 (46.0%): 273 (54.0%)	0.52
peak CTNI	0.10 [0.17]	0.10 [0.10]	0.16
D-dimer	3.45 [505.93]	2.30 [11.63]	0.97
BUN	27.00 [32.50]	20.00 [13.00]	<0.01*
Creatinine	1.25 [0.70]	1.10 [0.40]	<0.01*
Blood sugar	116.00 [58.00]	116.00 [58.00]	0.56
Hb	12.97 ± 3.08	13.23 ± 2.26	0.40
HCT	40.40 [12.60]	40.63 [8.40]	0.84
MCV	88.00 [7.70]	86.00 [8.10]	0.08
RDW	50.05 [10.2]	46.90 [6.70]	<0.01*
WBC*1000	11.00 [7.30]	9.90 [4.90]	0.32
Neutrophil*1000	7.80 [6.79]	6.95 [4.33]	0.50
Lymphocyte*1000	1.35 [1.06]	1.70 [1.27]	<0.01*
PLT*1000	175.00 [149.00]	197.00 [97.25]	0.04*
MPV	10.00 [1.90]	9.80 [1.30]	0.16
SBP	110.00 [30.00]	120.00 [30.00]	<0.01*
HR	98.00 [26.50]	99.00 [30.00]	0.99
RR	20.00 [6.00]	20.00 [6.00]	0.44
BT	37.00 [0.30]	37.00 [0.30]	0.71
TG	113.00 [72.50]	117.00 [71.00]	0.46
Cholesterol	146.00 [60.50]	165.50 [59.75]	<0.01*
LDL	80.00 [48.00]	102.00 [52.00]	<0.01*
HDL	31.00 [19.00]	35.00 [14.00]	0.04*
HTN	25 (61.0%)	201 (39.7%)	<0.01*
DM	12 (29.3%)	90 (17.8%)	0.06
Smoking	8 (19.5%)	65 (12.8%)	0.22
Ratios			
Cholesterol/Neutrophile*1000	17.75 [15.24]	23.51 [17.45]	<0.01*
LDL/Neutrophile*1000	9.47 [9.74]	14.60 [13.42]	<0.01*
HDL/Neutrophile*1000	3.37 [3.36]	4.97 [3.81]	<0.01*

The numeric data are presented in median [IQR] or mean ± SD, based on the normality of distributions and nominal data are presented in number (percentage). CTNI: Cardiac Troponin I; BUN: Blood urea nitrogen; Hb: Hemoglobin; HCT: Hematocrit; MCV: Mean corpuscular volume; RDW: Red cell distribution width; WBC: White blood cells; PLT: Platelets; MPV: Mean platelet volume; SBP: Systolic blood pressure; HR: Heart rate; RR: Respiratory rate; BT: Body temperature; TG: Triglycerides; LDL: Low-density lipoprotein; HDL: High-density lipoprotein; HTN: Hypertension; DM: Diabetes mellitus.

**Table 2:** Factors associated with in-hospital mortality in multivariant logistic regression analysis

**Characteristics**	**B (95%CI)**	***P*** ** value **
SBP	1.011 (0.994 – 1.029)	0.207
HTN	2.093 (0.865 – 5.067)	0.101
DM	1.080 (0.402 – 2.901)	0.879
TG	1.000 (0.995 – 1.004)	0.973
Cholesterol	1.010 (0.972 – 1.049)	0.624
LDL	1.012 (0.964 – 1.062)	0.641
HDL	0.962 (0.889 – 1.041)	0.340
Hb	0.951 (0.791 – 1.142)	0.589
RDW	0.999 (0.979 – 1.020)	0.937
Neutrophil*1000	1.000 (1.000 – 1.000)	0.715
Lymphocyte*1000	1.000 (1.000 – 1.001)	0.102
PLT*1000	1.000 (1.000 – 1.000)	0.399
peak CTNI	1.181 (0.634 – 2.199)	0.601
BUN	0.979 (0.952 – 1.007)	0.143
Creatinine	0.866 (0.579 – 1.294)	0.482
Cholesterol/Neutrophile	1.013 (0.766 – 1.339)	0.930
LDL/Neutrophile	0.902 (0.639 – 1.274)	0.559
HDL/Neutrophile	1.382 (0.808 – 2.362)	0.238

CTNI: Cardiac Troponin I; Hb: Hemoglobin; RDW: Red cell distribution width; SBP: Systolic blood pressure; HR: Heart rate; RR: Respiratory rate; BT: Body temperature; TG: Triglycerides; LDL: Low-density lipoprotein; HDL: High-density lipoprotein; HTN: Hypertension; DM: Diabetes mellitus; PLT: Platelets; BUN: Blood urea nitrogen.

**Figure 1:**
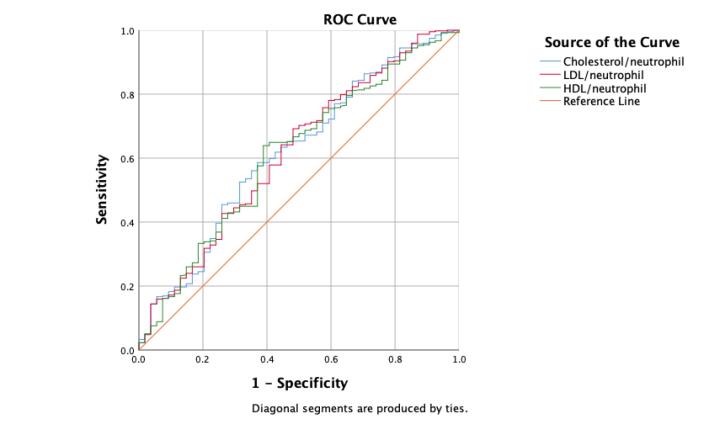
The ROC curves for predicting short-term survival for the variables investigated in this study. LDL: Low-density lipoprotein; HDL: High-density lipoprotein

 Based on our findings, cholesterol/neutrophil*1000 with a cut-off value of 22.014 can predict short-term survival in PTE patients with 56.7% sensitivity and 61.3% specificity (AUC: 0.71 [95%CI: 0.62-0.80], *P* value < 0.01). Also, LDL/neutrophil*1000 with a cut-off value of 10.909 (sensitivity: 69.3%; specificity: 51.9%; AUC: 0.72 [95%CI: 0.63-0.81], *P *value < 0.01) and HDL/neutrophil*1000 with a cut-off value of 4.150 (sensitivity: 61.9%; specificity: 58.1%; AUC: 0.59 [95%CI: 0.52-0.67], *P *value < 0.01) can predict short-term survival in patients with acute PTE, too. Between the mentioned parameters, LDL/neutrophil, cholesterol/neutrophil, and HDL/neutrophil were found to be the best prognostic factors for the short-term survival of patients with PTE. ROC curves for each of the discussed ratios are presented in Figure 1.

## Discussion

 This study was designed to assess the predictive value of lipid-to-neutrophil ratios for the short-term survival of patients with PTE. We concluded that cholesterol/neutrophil, LDL/neutrophil, and HDL/neutrophil ratios have a moderate predictive power in the short-term prognosis of patients with PTE. However, this correlation lost its significance in multivariant logistic regression. This could be duo to the presence of interaction with other indicators or the presence of many predictor variables, assuming a suitable sample size and sufficient power.

 PTE has an incidence rate of approximately 60-70 per 100 000, among the general population and if untreated, its mortality can be as high as 30%.^16-18^ Because most PTE patients ultimately die within the first hours of presentation, early diagnosis and having an insight into its possible prognosis are of paramount importance.^19^ Few scores are used to determine the prognosis of PTE patients like PESI and simplified PESI score, Geneva score, and 2014 European Society of Cardiology (ESC) mode, however recent studies indicate that the Geneva risk score and 2014 ESC model are not reliable to identify the high-risk PTE patients. Moreover, although the PESI score can be reliable for identifying the low risk of early mortality in PTE patients, clinicians still question its ability to identify the high risk of early mortality in them.^20-22^ Recent studies have indicated several laboratory parameters including brain natriuretic peptide (BNP), N-terminal-proB-type Natriuretic Peptide (NT-proBNP), interleukin (IL)-6, IL-8, heart-type fatty acid binding protein (H-FABP), troponin and myoglobin as a possible prognostic factor for PTE patients,^17,18^ however, accessibility, availability, and cost-effectiveness limit their use in the clinical practice. In this condition, the widely available and accessible parameters such as CBC. diff findings and lipid profile are suggested as an appropriate predictive factor for mortality in PTE patients. Studies have reported ratios like monocytes to HDL ratio or neutrophil to lymphocyte ratio as probable prognostic factors for PTE.^23,24^ This study found cholesterol/neutrophil, LDL/neutrophil, and HDL/neutrophil ratios as possible predictive factors for IHM in PTE patients.

 Recently, a new concept called “lipid paradox” has been introduced which means that a lower rate of lipid parameters like serum TC, LDL, and TG have a significant relationship with a higher rate of IHM in cardiovascular diseases like acute coronary syndrome and myocardial infarction.^25^ From the pathophysiological point of view, the basis of the thrombotic process is inflammation leading to oxidative changes that can decrease cholesterol synthesis. Also, acute-phase reactants can increase cholesterol uptake by hepatocytes.^26,27^ In addition, recent studies on mice have shown that HDL and Cholesterol have an important role in lung normal function and have a vital role in the regulation of pulmonary inflammatory response after tissue injury.^28,29^ Studies also demonstrate a correlation between venous Thromboembolism (VTE) and inflammation.^30^ On the other hand, HDL can protect endothelial cells against inflammation and oxidative stress by preventing monocyte flow to the arterial wall, which reduces the expression of CD11b on monocyte and endothelial molecules and prevents the adhesion of monocytes to the endothelial wall.^31-33^ Finally, TGs are known as important energy sources for peripheral organs. An increase in acute phase reactions increases the function of lipoprotein lipase that breaks down circulating TGs and results in lower TG levels.^34^ In a study by Karatas et al serum TC, LDL, HDL, and TG levels were significantly lower in deceased patients when compared to the surviving PTE patients.^12^ In another study by Avci et al, serum levels of HDL were also significantly lower in deceased PTE patients.^23^ In this study also the serum TC, LDL, HDL, and TG levels were significantly lower in PTE patients who died during their hospitalization.

 On the other hand, studies indicate that leukocyte count could be related to fibrinogen, factor VII, and factor VIII levels and can cause local thrombogenic activity.^35,36^ Moreover, stimulated neutrophils may be responsible for vascular injury due to increased cytokines secretion^37^, which can be a result of severe hypoxia caused by pulmonary artery obstruction and an increase in neuro-hormone and adrenergic system activity. This reaction may aggravate thrombosis and the severity of the disease in patients.^38^ In a study by Kayrak et al WBC, neutrophil, and lymphocyte counts were significantly higher in deceased PTE patients in comparison to survivors.^39^ Another study by ÇAVUŞ et al also indicates the same result.^24^ In this study, lymphocyte count was significantly higher in the mortality group however WBC and neutrophil count didn’t have a significant difference between the death and alive groups.

 Recent investigations suggested the neutrophil to HDL ratio as a prognostic factor for the severity of coronary arteries stenosis,^40^ clinical outcomes of patients with MI^41^, and all-cause and cardiovascular mortality in the general population.^42^ As one of the first tries, we investigated the relationship between lipids to neutrophil ratios and IHM of PTE patients in a great cohort and we found cholesterol/neutrophil, HDL/neutrophil, and LDL/neutrophil ratios as good predictors of short-term survival in PTE patients. Some limitations may affect our findings. One of them is the retrospective design of the study and the second one is the lack of long-term follow-up in the study. Also, this study was not able to exclude patients who had a drug history of statins. Furthermore, it’s recommended to compare the suggested ratios in this study with other risk scores such as PESI in future studies. Considering the impacts of acute PTE-related inflammation on lipid profile,^43,44^ this study cannot ensure that measured lipid levels and calculated ratio represent patients’ chronic lipid status. In addition, this study suggests larger prospective multicenter studies to evaluate the possible role of serum lipid profile and cholesterol/neutrophil ratio in the prognosis of PTE, while considering the possible confounders such as body mass index (BMI) and recent weight loss. In case of approval of the findings of this investigation in future studies, we suggest that the mentioned ratio could be included in the risk stratification algorithms of PTE.

## Conclusion

 PTE patients with cholesterol/neutrophil*1000 < 22.014, LDL/neutrophil*1000 < 10.909, and HDL/neutrophile*1000 < 4.150 have a higher rate of IHM, which suggests these ratios are a good prognostic factor for predicting short-term mortality in PTE patients. Measurement of lipid to neutrophil in the first 24 hours of hospitalization may be a valuable marker for determining the early prognosis of PTE.

## Acknowledgments

 The research protocol was approved and supported by the Student Research Committee, Tabriz University of Medical Sciences (grant number: 70814). The authors also would like to appreciate the cooperation of the clinical research development unit of Shahid Madani Hospital, Tabriz, Iran.

## Competing Interests

 None.

## Ethical Approval

 The study process was reviewed and approved by the ethics committee of Tabriz University of Medical Sciences, according to the declaration of Helsinki (ethics code: IR.TBZMED.REC.1401.1011).
